# Re-inhabiting one’s body: A pilot study on the effects of dance movement therapy on body image and alexithymia in eating disorders

**DOI:** 10.1186/s40337-020-00296-2

**Published:** 2020-05-11

**Authors:** Maria Savidaki, Sezin Demirtoka, Rosa-María Rodríguez-Jiménez

**Affiliations:** 1 Psychology Department, Autonomus University of Barcelona, Plaça Cívica, Barcelona, 08193 Spain; 2 Universidad Europea de Madrid, c/Tajo, s/n, Villaviciosa de Odón, Madrid, 28670 Spain

**Keywords:** Eating disorders, Dance movement therapy, Body image, Alexithymia, Emotions, Quantitative, Qualitative, Controlled trial

## Abstract

**Background:**

Body image disturbance and alexithymia are two core aspects of Eating Disorders (EDs). However, standard treatments for EDs do not include specific techniques to approach these issues on a bodily level. This pilot study evaluated the effects of a Dance Movement Therapy (DMT) intervention on body image and alexithymia in patients with EDs, and also explored their experience of the therapeutic process.

**Method:**

14 patients with EDs were recruited from a private clinic. Seven were assigned via quasi-randomization to the DMT group and the others (*n* = 5) continued their treatment as usual. The length of the intervention was 14 weeks. All participants completed the Multidimensional Body Self Relations Questionnaire (MBSRQ) and the Toronto Alexithymia Scale (TAS-20) at the beginning and at the end of the intervention. Additionally, the DMT group wrote reflective diaries about their experience at the end of each session, which were analyzed using qualitative methods.

**Results:**

Between the pre- and post-intervention, the participants of the DMT group significantly improved in Body Areas Satisfaction (effect size: 0.95) and Appearance Evaluation (effect size: 1.10), and they decreased significantly in Appearance Orientation (effect size: 1.30). A decrease in Overweight Preoccupation was observed (effect size: 0.75), however this was not statistically significant. The control group did not show significant changes in any of the MBSRQ subscales. Neither the DMT group nor the control group improved significantly in the alexithymia scores. The qualitative analysis revealed valuable insights into the participants’ processes throughout the sessions. In general, participants received the DMT intervention positively. They reported improvements in their mood states and an increase in their self-awareness. They also appreciated the relationship with the group and the therapist.

**Conclusion:**

These results indicate that DMT might be a complementary treatment option for EDs, as it may be able to address body image issues more effectively than verbal therapies. More studies with larger samples are needed to confirm these promising preliminary results.

## Plain english summary

The effects of a 14-week DMT intervention on body image and alexithymia were tested in 12 patients with EDs. The DMT group (*n*=7) improved in various aspects of body image after the treatment, while no change occurred in the control group (*n*=5). Both groups continued to receive the standard treatment offered by the Clinic. Neither of the groups improved significantly in alexithymia scores. The qualitative analysis revealed that participants were satisfied with this intervention. According to this analysis, the DMT sessions increased their self-awareness, improved their mood states, and promoted the building of meaningful relationships. Taken together, the findings suggest that DMT could contribute to the treatment of EDs on various levels.

## Background

The prevalence of Eating Disorders (EDs) is increasing worldwide [1]. Dropouts [2] from eating disorder therapy and full relapses are common [3]. Comorbidity with other psychological disorders and health issues pose a challenge for professionals and require multidisciplinary treatments [4]. Widely used specialized treatments for EDs mostly focus on cognitive-behavioral aspects and weight restoration [5]. However, randomized controlled trials have generated contradictory results regarding the effectiveness of those treatments [6]. Furthermore, patients with EDs are often not content with the treatment they receive according to a review of qualitative research [7]. In light of these findings, many authors have underlined the need for novel approaches to treat EDs [8, 9].

One of the core characteristics and a risk factor of EDs is body image disturbance, which has implications on cognitive, emotional and behavioral levels [10]. A person’s body image is formed since birth. The first experience of the self is through proprioception, i.e. sensations that arise from within the body, which develops by a person’s perceptions while relating to others and influences the emotions, attitude, psychological functioning, and self-value [11]. Cash describes it as an “inner view” which refers to the multidimensional embodied experience related mostly to one’s physical appearance [12]. Patients with EDs, especially those with anorexia nervosa, tend to focus on shape, weight, and food perhaps as a way of ignoring thoughts and emotions that provoke anxiety [13]. Thus, they consider their bodies with disdain and often fail to recognize their bodily needs [14].

Another personality construct that is common in individuals with EDs is alexithymia [15], which literally means “no words for emotions”. Alexithymia is characterized by an externally focused cognitive style and a difficulty in differentiating between feelings and bodily sensations [16]. This personality construct is also related to poor self-regulation [17]. As people with alexithymia face difficulties in mentalizing the emotional state of others and their own, they can be perceived as non-empathetic and experience difficulties in social relations [18]. Alexithymia is also associated with poor creative capacity and a limited ability of symbolization and play [19]. The absence of consciousness of the internal experience and the difficulty of trusting the perception of one’s emotions, sensations, and reflections are related to the sense of poor self-efficacy that patients with EDs face, as a result of not being able to resolve the conflict between the illusion of perfectionism, their actual potentials, and the expectations of others [20].

The development of body image and socioemotional functioning starts on a nonverbal level in infancy [21]. The infant first experiences its body through the tactile experience provided by the caregiver [11]. The empathetically attuned interactions with the caregiver provide a secure holding which defines the infant’s inner state by offering psychological and physical soothing, something that is the critical basis for secure attachment [22]. The term of psychesoma as defined by Winnicott [23] has its origins in the holding phase. When the handling is good enough the psyche can indwell in the body (soma) providing a sense of internal reality and structure of the self [23]. On the contrary, misattunement with the needs of the infant can arouse an inner confusion and a detachment between the subjective and physical elements of emotions, which is at the core of ED’s pathology [20]. The foundation of emotion-regulation and self-regulation is based on the secure attachment with the caregiver [24]. It is well established that attachment insecurity is an important risk factor for the development of EDs and a predictor for poor outcomes [25]. One study has found that improvement in attachment insecurity led to a decrease in interpersonal problems in patients with EDs [26]. Moreover, a review of three meta-analytic procedures found that secure attachment style is a crucial predictor for beneficial psychotherapeutic outcomes [27]. These findings indicate that treatment models for EDs should address attachment related disorders. Considering that the development of attachment is rooted in a pre-verbal phase, such a treatment model can make use of a bodily, non-verbal component.

The importance of bodily experiences and body memory for the psychological functioning has been shown by cognitive neuroscience studies [28]. Recent research in interpersonal neurobiology considers the brain in the whole nervous system, existing in the whole body [29]. These findings have encouraged psychotherapists to investigate the role of the body in verbal psychotherapies [30]. One bodily based approach is Dance Movement Therapy (DMT), which explores and uses movement in a therapeutic way with a focus on self-awareness, in order to promote the psychophysical integration of the individual [31]. This psycho-dynamic model, forms part of the Creative Art Therapies, in which music, art, and drama therapy are also included [32]. Some of the core characteristics among others for this group of therapies are the possibility of active participation, the self-expression, the emphasis in imagination, and the mind-body connections [33]. In particular, DMT was formed by dancers and psychotherapists in the 1940s, who realized the therapeutic impact of the artistic expression in mental health patients [33]. It is a form of psychotherapy based on embodiment and enaction [34]. The core basis of DMT is the innate connection between body and mind [34]. The unintentional expression through movement is fostered, which permits the enactment of unconscious ideas and emotions that often cannot be put into words [35]. This not only discharges psychological or corporal tensions but, simultaneously, can help the individual become aware of his/her emotional resources and strengths [36]. This therapeutic approach is both non-verbal and verbal [37, 38]. The non-verbal interactions can promote insights regarding the participants’ behavior, beliefs, relationship patterns, and emotional apparatus [39]. The verbal communication of thoughts and emotions has a fundamental role in the therapeutic process, and gives meaning to the symbolic aspects of the movement [33]. The therapist-patient relationship is a central component in DMT approximation. The DMT therapist uses specific tools of movement analysis in order to observe and interpret the participants’ body language. Building upon these observations, techniques such as attunement and mirroring are applied, which provide a link between attachment and non-verbal behavior [40]. The creative play and the transitional object concepts are also included in the therapeutic process [41]. Those means help the improvement of embodied relationships and give meaning to the movement [42]. The emotional state of the patients, as well as their early misattuned experiences with the caregiver, can be experienced, explored, felt and understood by the patient [34] through a deep attunement with the therapist on an emotional and bodily level [43]. These moments of meeting serve as a healing emotional experience [44]. The union between body and mind, as opposed to the mind-body dualism, assumes the principle that “the body reflects the mind and the mind reflects the body” [43]. Following up on this principle, our expectation is that changes in movement patterns can cause a modification in the internal comprehension of the individual and vice versa [45]. Other authors like Jung have mentioned the importance of the symbolic part in the therapeutic process [46]. The symbolic part of movement and the communication via movement metaphors, which is a core feature of DMT, can be helpful for alexithymic patients as it can promote the exploration of one’s emotional state and facilitate the therapeutic process [35]. The bodily and sensorimotor components of empathy are indicated by studies in neuroscience [47] so that, the psychotherapeutic use of body movement in DMT can enhance empathy among alexithymic individuals. In the same way, DMT can help people who suffer from EDs to experience themselves in a more complete and embodied way, since they are encouraged to make connections between the cause factors of EDs and the corporal manifestations of them [48], gaining body awareness [34].

Significant improvements have been associated with the use of DMT as an adjunct in the treatment of a wide variety of disorders, including anxiety, depression, schizophrenia, dementia [49, 50]. Improvements included an increase in quality of life ratings, as well as interpersonal and cognitive skills [50]. Such outcomes were comparable to pharmacological interventions and verbal psychotherapies [49, 50]. Some studies have found DMT interventions to improve the body image in patients with obesity [51], cancer [52] and fibromyalgia [53]. Apart from those findings, a major amount of research regarding DMT is focused on case studies and qualitative research which could be explained by the characteristics of DMT as the emphasis is put on the subjective experience, therapeutic relationship, personal development and change of the person as well as on the creative and aesthetic part of the therapeutic process [54]. Considering the alexithymic aspects and body image issues in EDs, treatment should promote the physical and psychic integration of the individual, which is why DMT is a potentially adequate approach for patients with EDs.

The proposed therapeutic intervention was designed for the needs of a group, which consisted of patients with EDs. As it was mentioned in previous studies, difficulties in accessing self and body awareness as well as the resistance towards movement exploration [55] and negative body experiences were identified [56]. The guided imagery by Reddemann [57] was used as a mediator to facilitate the connection of the participants with the body and the self in movement and to enrich one’s movement vocabulary.

The current research is a pilot study. It aims to explore a DMT intervention that could complement the already existing ED treatment in an attempt to provide a more holistic and effective approach. Due to the theoretical considerations, research findings and the mixed nature of the sample, a transdiagnostic perspective was followed [58]. Firstly, changes in the body image and alexithymic traits as a result of the proposed intervention were explored. Secondly, as the DMT intervention on the ED population is rarely investigated, a qualitative analysis was included in the research, to develop a better understanding of the patients’ experience. To the authors’ opinion, a mixed study that integrates quantitative and qualitative analysis can provide a more holistic view.

## Method

### Participants

The participants were 14 young women who attended a private day clinic in Spain, specializing in the treatment of EDs. Participants’ ages ranged from 14 to 32 (M = 20.28, SD = 5.91). BMI ranged from 14.03 to 24.69 (M = 18.94, SD = 3.56). Criteria of inclusion were: receiving standard treatment from the Clinic and having been diagnosed with an ED according to the Diagnostic and Statistical Manual of Mental Disorders, Fifth Edition (DSM-5) [59]. Exclusion criteria were: comorbidity with personality disorders and/or the administration of medications, as those could modify the movement qualities of the participants. These criteria were aiming at creating a more homogeneous group in order for the obtained results to be as representative as possible for the ED population. One participant was excluded from the study as she did not complete the predetermined criteria. The final sample size was determined by the number of the patients in the Clinic who fit these criteria.

### Setting and procedure

The Clinic where the study was conducted offered mainly a cognitive behavioral treatment approach. The weekly schedule consisted of three group therapies, six psychoeducational sessions, and one individual therapy session for each patient. The number of sessions that each patient received was adapted according to her needs. The Clinic was informed of and approved the structure, content, and procedures related to the DMT intervention, as well as the possibility of publication of the obtained research data. Written approval was provided to the authors by the ethical committee of the Clinic. All participants signed a consent form, agreeing to the anonymized use of self-report questionnaires in the current study. The DMT group signed a second consent form regarding the anonymized use of reflective diaries that were used in this investigation. As part of the consent forms, they were provided with detailed information about the framework of the investigation and the research questions. The groups were quasi-randomized, as a professional from the Clinic assigned the participants to the DMT group or the control group depending on their timetable. The DMT group was semi-open; new participants could join the sessions once they entered the day clinic and they had to leave the group when they were released from the Clinic. Twelve sessions were conducted over a 14-week period. Participation was optional. Both, intervention and control group, continued their treatment in the Clinic as usual. Self-report questionnaires were first completed prior to the participants’ entry to the DMT sessions, and again within four weeks from the last session. The control group followed the same procedure. The research, as well as the interventions, were part of the Master’s training in Dance Movement Therapy, which runs under the Department of Psychology of the Autonomous University of Barcelona. The sessions were facilitated by a dance movement therapist in training, who received clinical supervision from the University during the entire process. The research was supervised by a researcher from a different university, who specialized in DMT and EDs. Another dance movement therapist in training participated in the data analysis.

### Intervention

The DMT sessions were based on Chace’s model [60], and guided imagery according to Reddemann [57]. Each session was divided into 6 parts: check-in, warm-up, guided imagery, exploration in movement, writing, check out. During check-in, the participants were invited to share their current emotional state. Based on that, the therapist guided the warm-up in order to activate the body and prepare the participants for the main part of the session. During the guided imagery part, the participants were provided with self-regulatory techniques in order to remain in the “here and now” and to reduce stressful thoughts. The aim was to experience feelings of safety and comfort as well as to explore one’s own emotional resources. During the exploration in movement, themes that have emerged in the check-in, warm-up or guided imagery part could be accessed in depth. The creative process aimed to transform participants’ inner experiences into external realities. The objectives and dynamics proposed in the movement exploration part are shown in Table 1. Afterwards, the participants had to write down their reflections upon the process. In addition, to the investigation purposes, this part also aimed at reintegrating the lived experience. During the check-out, the participants were given the opportunity to verbalize and share their experiences.

**Table 1:** Dynamics of the DMT sessions

**Objective**	**Intervention**	**Examples**
Connection with one’s	Breathing exercises	–Participants observe the impact of deep breathing in their bodies.
self		– Breathing in circle: A touches her stomach and the back of B,
		B touches her stomach and the back of C, and so on.
		Use of light and sustained movements.
		– Participants observe their own breathing and the one of the
		other’s.
Body awareness	Exploration with	–Participants move an object through different parts of the body
	objects	and observe the impact of this contact and the difference between
		the parts of the body that have not been touched.
		Use of light, free, direct, and sustained movements.
Interaction among the	Movement in pairs	– Participants work in pairs:
participants		– A touches B and B responds by accepting or rejecting the touch.
		–A touches B and B moves that part of the body. Light, indirect movements.
		– A touches B and B resists by putting force in that part of the body. Strong,
		direct movements.
Exploration of limits	Exploring the space	– Participants use objects (e.g., wool, elastics, fabrics) to visualize
		their personal space.
		- Exploration with elastics: First each participant uses an elastic
		band to integrate her muscles in movement and explore her limits.
		As a next step, they continue this exploration in pairs, sharing
		one elastic band. Use of different movement qualities.
Group cohesion	Group games	– First in a circle, participants pass each other the ball in a certain
		order, saying the name of the person that the ball is directed to.
		They continue in the same order without saying the names.
		Movement and music can be introduced and the pace can be varied.
		– Use of fabrics: After experimenting individually with a fabric,
		participants get together in small groups and create a group dance.
Empathy	Movement exploration	-One participant chooses a place in the room and closes her eyes
		(optional). Other group members slowly approach her and offer the
		touch they think she needs (e.g., holding a hand, gently touching the
		head). This activity can be proposed once there is sufficient
		confidence in the group. Participants are advised to say no if they
		feel uncomfortable. Use of light, direct, and sustained movements.
Reflection	Group circle	– After experimenting with different movement qualities (e.g. fluid,
		robotic) participants are invited to share their associations with
		these, and possibly relate them to their personal lives and ED
		symptoms.
		– Participants use different objects to transmit to the group what
		they felt during the session.

Note: The references in movement qualities, derived from Laban movement analysis that is used in DMT. They are related to the indulgence or the resistance of the participant and may indicate the way of relating with the other.

The sessions lasted 90 minutes. For the check-in and check-out (oral verbalization) 10’ was given for each. The warm-up took 10’. For the guided imagery part the participants had 20’, followed by the movement exploration (30’). Finally, the writing part (personal reflections) took 10’. The time could be varied according to the general group status.

The intervention was divided into the following phases: in the first four sessions (1-4), the focus was on creating an atmosphere of safety and trust which played a crucial part throughout the whole therapeutic process. The connection of the participants with their own self was also promoted from the very first encounters. From the 5^th^ to the 10^th^ session the focus was shifted to a facilitation of the group cohesion. Participants were invited to explore their movements in pairs which led to empathetic interactions among the group members. The main aim of this phase was to promote self- and body-awareness as well as creating a safe atmosphere where the participants could share their experiences. During the last two sessions (11-12) the focus was shifted in preparing the participants for the upcoming closure of the DMT intervention. The therapist brought the participants’ attention back to the connection with their own self and with others, as a way to support their internal reorganization.

The sessions were conducted in a spacious room of the Clinic, where participants could move freely. Objects played a significant part in the therapeutic process. They were used in the majority of the sessions in order to facilitate the contact of the individuals with their bodies, as well as their interaction with the other participants. The objects used were: elastics, different forms of fabrics, small hard (tennis) balls and soft balls, balloons, ribbons, scarves, paper and crayons.

### Qualitative measures

Participants of the DMT group were invited to keep reflective diaries in order to obtain a better understanding of their processes. Each participant was given a notebook and wrote in it freely at the end of each session. Due to the exploratory nature of this study, no specific instructions were given about the content of these texts. The therapist told the participants to write about whatever they wanted, in order to minimize possible biases. Reflective diaries were transcripted, and codified. Successive readings of the material were made looking for themes. This process was repeated until saturation was reached, following a classical content analysis [61]. Investigator triangulation was applied by including a second researcher who independently and blind to the intervention process, carried out the content analysis. The whole analysis was made once the therapeutic process was over. Nvivo 12 software was chosen for the analysis [62]. The extracts from the reflective diaries presented in the current research were translated to English by one of the authors. The trainee therapist’s observation diary was also included as another source of data collection. Information from the therapist’s observation diary was used to illustrate some specific moments during the sessions.

### Quantitative measures

Two self-report questionnaires were used for the quantitative part of this study.

Toronto Alexithymia Scale (TAS-20) [63, 64]. The Spanish version [65] of the TAS-20 was used to measure alexithymia. Items are rated on a 5-point Likert scale. It consists of three subscales: Difficulty Identifying Feelings, Difficulty Describing Feelings to others, Externally Oriented Thinking. The scales have adequate reliability (*α*=.794, *α*=.732, *α*=.613 respectively) and adequate convergent validity.

Multidimensional Body-Self Relations Questionnaire (MBSRQ) [66]. The 45-item Spanish adaptation of the MBSRQ questionnaire was used to measure body image [67]. The subscales Appearance Evaluation (satisfaction or dissatisfaction with one’s physical appearance), Appearance Orientation (importance placed on one’s physical appearance and effort put in maintaining one’s looks), Body Areas Satisfaction (satisfaction or dissatisfaction with specific body areas e.g., stomach, legs, etc.) and Overweight Preoccupation (ideas regarding fat anxiety, weight vigilance, etc.) were used in this pilot study. Cronbach’s *α* were calculated for the MBSRQ subscales used in the current study. All the subscales were found to have adequate internal consistency (*α*=0.937, *α*=0.867, *α*=0.929, *α*=0.900).

### Data analysis

Following the confirmation of the parametric distribution of the quantitative variables, paired-samples t-tests were conducted to compare differences pre- to post-intervention in each group. Cohen’s d effect sizes were calculated. For not normally distributed data, Wilcoxon Signed Rank test was applied to compare pre- and post-intervention scores in each group and Rosenthal’s r effect sizes were calculated. Values of 0.2, 0.5, and 0.8 are interpreted as small, medium, and large effect sizes respectively [68]. Given the small sample size, both statistically and clinically significant results were taken into account. Statistical Package for the Social Sciences (SPSS), version 20.0 for Windows [69] was used for statistical analysis.

## Results

### Demographic summary

Of the 14 young women who were recruited for the study, 7 were assigned to the DMT group and 7 to the control group. They were all high school or university students of Spanish nationality. The DMT group consisted of 4 EDNOS and 3 AN patients aged between 14 and 32 (M = 20.1, SD = 5.9). The BMI of the DMT group ranged from 14.03 to 24.69 (M = 19.82, SD = 4.37). The length of time since their admission to the Clinic at the beginning of the intervention ranged from 1 to 23 weeks (M = 13.43, SD = 9.11). Two participants from the control group did not complete the follow-up questionnaires, so that they had to be excluded from the sample. The final control group (*n* = 5) consisted of 2 AN, 2 EDNOS and 1 BN patients aged between 17 and 23 (M = 20.3, SD = 2.5). The BMI of the control group ranged from 17.33 to 22.9 (M = 19.07, SD = 2.28). The length of time since their admission to the Clinic at the time of the first data collection ranged from 24 to 59 weeks (M = 42, SD = 15.7). T-tests showed that the two groups were homogeneous concerning their age (t=0.21; *p*=0.84) and BMI (t=0.19; p=0.83), however not regarding the length of time at the Clinic. The participants of the control group had been in the Clinic for significantly longer period at the time of the first data collection, t=4.0, p=0.02. The implications of this difference for the current study are considered in Limitations.

The 7 participants in the intervention group completed a minimum of 4 and a maximum of 11 sessions (M=7.71, SD=2.93). One participant completed 11, two completed 10, one completed 9, two completed 5 and one completed 4 sessions. As a result, an average of 4 to 5 participants were involved in each DMT session.

### Quantitative outcomes

The mean scores for the MBSRQ subscales are presented in Table 2. Prior to intervention, for Appearance Evaluation participants in the DMT group were more than 1 SD below the norm (M = 3.36, SD = 0.87). For Appearance Orientation they were within 1 SD from the norm (M = 3.91, SD = 0.60). For Body Areas Satisfaction they were more than 1 SD below the norm (M = 3.23, SD = 0.74). For Overweight Preoccupation they were more than 1 SD above the norm (M = 3.03, SD = 0.96) [66]. As all the MBSRQ subscales were normally distributed in both groups, paired samples t-tests were conducted to compare the pre- and post-intervention scores in each group. As can be seen in Table 2, the DMT group increased significantly in Appearance Evaluation and Body Areas Satisfaction, and decreased significantly in Appearance Orientation pre- to post-test. Large Cohen’s d effect sizes were observed for each of these variables. There was a tendency to decrease in Overweight Preoccupation which was not statistically significant, however a medium to large effect size was observed. Post-test mean scores of the DMT group for Appearance Evaluation, Body Areas Satisfaction, and Overweight Preoccupation were within 1 SD from the norm. No significant differences were found in the control group between pre- and post-test in any of the subscales.

**Table 2:** Results of paired samples T-Test for pre- and post-intervention for MBSRQ subscales in DMT and Control groups

		**Descriptive statistics**	**T-test**			
**MBSRQ**	**Group**	**Pretest**	**Posttest**	**t (df)**	**p**	**Cohen’s d**
**Subscales**		**Mean (SD)**	**Mean (SD)**			
Appearance	DMT	2.34 (1.09)	2.94 (0.81)	-2.51 (6)	.046	-.95
Evaluation	Control	2.80 (0.97)	3.04 (0.89)	-1.04 (4)	.358	-.46
Appearance	DMT	3.92 (0.79)	3.41 (0.73)	3.44 (6)	.014	1.30
Orientation	Control	3.43 (0.88)	3.46 (0.73)	-.23 (4)	.831	-1.02
Body Areas	DMT	2.24 (0.80)	2.76 (0.71)	-2.91 (6)	.027	-1.10
Satisfaction	Control	3.07 (1.27)	2.93 (1.12)	.75 (4)	.494	.34
Overweight	DMT	4.29 (0.76)	3.64 (0.56)	2.00 (6)	.093	.75
Preoccupation	Control	2.70 (1.92)	2.80 (1.82)	-.41 (4)	.704	-.18

When a mixed-effects ANOVA model was applied, results for time effect were consistent with previous results for Appearance Evaluation (F=5.93; $\eta ^{2}_{p}=0.37$; *α*=0.04) and Appearance Orientation (F=5.54; $\eta ^{2}_{p}=0.36$; *α*=0.04). For Body Areas Satisfaction time effect was not significant (F=2.23; $\eta ^{2}_{p}=0.18$; *α*=0.17). Regarding the interaction effect of time and group, significant changes appeared in Appearance Orientation (F=6.90; $\eta ^{2}_{p}=0.41$; *α*=0.03) and Body Areas Satisfaction (F=6.32; $\eta ^{2}_{p}$=0.39; *α*=0.03). The interaction effect for Appearance Evaluation was not statistically significant (F=1.09; $\eta ^{2}_{p}=0.09$; *α*=0.32). Finally, for the Overweight Preoccupation the ANOVA model was consistent with the previous analysis. Neither time (F=1.55; $\eta ^{2}_{p}= 0.13$; *α*=0.24) nor interaction effects (F=2.90; $\eta ^{2}_{p}=0.23$; *α*=0.12) were significant.

Prior to the intervention, alexithymia scores ranged between 47 and 71 in the DMT group and 27 to 60 in the control group. According to the cutoff scoring, in the DMT group 4 participants did not display alexithymia, 1 displayed possible alexithymia and 2 displayed alexithymia. In the control group, 4 people did not display alexithymia, and 1 displayed possible alexithymia. As the data was not normally distributed, Wilcoxon Signed Ranks tests were undertaken to compare pre- and post-intervention results of the DMT and control groups (see Table 3). There was a slight tendency of decrease in the total score and the subscales of alexithymia in the DMT group, which was not statistically significant. Small Rosenthal effect sizes were observed in Difficulty Identifying Feelings and in Difficulty Describing Feelings to Others. On the contrary, the control group had a tendency to increase in the total score and the subscales of alexithymia. This increase was not statistically significant, however moderate effect sizes were observed in the Alexithymia total score, in Difficulty Identifying Feelings, and in Difficulty Describing Feelings to other, and a small effect size was observed in Externally oriented thinking.

**Table 3:** Results of paired samples T-Test for pre- and post-intervention for Alexithymia subscales in DMT and Control groups

		**Descriptive statistics**		**Wilcoxon signed rank test**		
	**Group**	**Pre Mdn**	**Post Mdn**	**z**	**p**	**Rosenthal’s r**
		**(IQR)**	**(IQR)**			
Alexithymia	DMT	49 (48-70)	46 (42-61)	-0.593	.553	-.16
Total Score	Control	35 (30-52.5)	50 (40-53)	-1.753	.080	-.55
Difficulty	DMT	21 (17-26)	20 (12-26)	-0.734	.463	-.20
Identifying Feelings	Control	9 (9 - 21.5)	13 (10-23)	-1.841	.066	-.58
Difficulty Describing	DMT	14 (13-20)	11 (10-19)	-1.156	.248	-.31
Feelings to Others	Control	10 (6.5-13.5)	13 (11.5-14.5)	-1.604	.109	-.51
Externally Oriented	DMT	18 (15-23)	17 (15-21)	-0.171	.865	-.05
Thinking	Control	14 (11-22)	19 (12.5-24.5)	-0.816	.414	-.26

Note: Cut-off scoring for Alexithymia total score goes as follows: <51 = non-alexithymia, 51-60 = possible alexithymia, >60 = alexithymia

### Qualitative outcomes

There were five domains that emerged from the analysis of the qualitative data. Within each domain, a number of themes are identified (see Table 4). Each theme is presented, followed by a selection of related extracts from the data. Due to space limitations only some of the evidences are presented to illustrate each category. The code of each quote indicates the code given to the participant and the number of the session when the text was written (e.g., P1S01 refers to Participant 1, Session 1).

**Table 4:** Qualitative analysis. Domains, themes, and sub-themes

**Domain**	**Theme**	**Sub-theme**
1. Emotion, mood,	1. Emotional states (7)	1.1.1. Patterns of mood changes
and alexithymia		during the sessions (6)
		1.1.2. Getting in touch / dealing
		with strong feelings (3)
	1.2. Alexithymia, connection, and disconnection (6)	
2. Body and movement	2.1. Joys of movement (7)	
	2.2. Difficulties related to DMT activities (7)	
	2.3. A pattern of activating during the session (6)	
3. Interpersonal aspects	3.1. Enjoyment of the interactions in the group (6)	
	3.2. Difficulties in relationships (5)	
4. Metaphors	4.1. “Safe place” (6)	
	4.2. The use of metaphor to describe a feeling (2)	
5. Reflections	5.1. Reflections on one’s process (3)	
	5.2. Looking back on the DMT sessions (4)	

Numbers in parentheses indicate how many participants shared this category

#### Domain 1: Emotion, mood, and alexithymia

**Emotional and mood states**

All the participants made attempts to identify and describe how they felt at the beginning, during or at the end of the session, related to both the present moment and the larger context of their lives. The level of detail of their descriptions varied, from “fine” to naming specific emotions to describe how they felt (the use of metaphors to describe a feeling is considered later, under the theme of metaphors).

Extracts 1

*“Today I was emotionally less calm and peaceful because of circumstances and decisions to take in the near future.” - P1S04*

*“Today I came in feeling indifferent and tired, I didn’t feel like doing anything after feeling not understood by my father yesterday.” - P5S08*

These participants described their moods briefly, comparing it to the previous days or weeks, or relating it to a situation that triggered these feelings. They mostly referred to anxiety vs. calmness, happiness vs. sadness and a lack of motivation.

**A pattern of mood shift during the session**

Most participants reported changes in their moods during the sessions.

Extracts 2

*“Today I’ve felt very calm. I’ve been through a couple of days with a lot of anxiety and the session helped me to lower it.” -P7S12*

*“I’m leaving the session more energetic and happy, feeling better than I was before. Motivated to do many things and endure whatever may come. Stronger.” -P5S10*

*“I am leaving the session a bit calmer and more relaxed (even though I don’t think it will last long, but I’m happy that even though for only a while I wasn’t stuck in my negative thoughts.)” -P6S10*

In these instances, the participants considered that the DMT sessions helped them reduce their anxiety, or offered a temporary relief from it. Moreover, they reported feeling more motivated after the sessions.

**Getting in touch with strong feelings**

Some participants described getting in touch with strong feelings that they probably were unaware of.

Extract 3a

*“I was surprised that I cried so much, so deeply and so intensely. I wasn’t aware that I was feeling all that so intensely.” - P6S07*

P6 expresses her surprise as she becomes aware of the strong feelings that were unknown to her before the session.

Extracts 3b

*“When A.*^1^* encouraged me to work through my anger, I started feeling it very strongly (even crying), first I thought it was anger towards myself, and then I realized it was anger towards the disease.” P2S08*

*“I felt moved by P2’s thing. Oddly enough my head was very lucid there, I was left with the urge to deal with my own anger because I feel that I have a lot and I quite identify the images, where they come from. But again, if I don’t have a clear secure place to turn to, perhaps it’s not a good idea to go to this anger because I might get stuck there […] I feel confused, my head is going to explode.” P6S08*

These extracts illustrate the attempts of these participants to accept and work through their anger, as well as their difficulties to stay with these feelings. P6 expresses how P2’s process inspires her to work through difficult feelings herself.

**Alexithymia, connection, and disconnection**

In addition to the quality of a feeling, a theme of recognition and awareness of the feelings emerged. Participants often included a dimension of being “connected” versus “disconnected” when talking about their mood states, which in clinical terms seem to describe their varying experiences of dissociation. The term dissociation is used loosely here in order to encompass the wide range of experiences of being more or less present in the body as described by the participants.

Extracts 4a

*“I don’t know exactly how I felt.” - P2S05*

*“Initial feeling: I’ve been emotionally shattered the whole week, without knowing exactly how I feel. Physically I feel well, but inside I’m nervous (because of the exams), tired of everything, sad…” - P5S03*

These participants recognized their difficulties in identifying feelings. P5 described an apathetic mood that had been going on for several days.

Extracts 4b

*“I felt very comfortable during the session. That’s for sure, but sometimes it was easy for me to disconnect and think about other things.” - P3S03*

*“At the beginning of the session, I found it very difficult to connect with myself, to stop connecting with my anxieties and unease, and to focus on the moment, on living in the moment.” - P5S11*

*“Today I feel absent, my head is somewhere else. I realize that everything is heavy. My body, my head, my soul, everything…” - P6S08*

These extracts show the variety of meanings that connection and disconnection have for the participants. Both P3 and P5 mention having difficulties to live in the present moment and an urge to think of other things. For P3, this seems to enable her to escape from current stimuli *“it was easy for me to disconnect”*, while for P5 getting into contact with the present is overwhelming. In general, these extracts present a tendency of these individuals to live more in their minds rather than in their bodies. In the last quote, as P6 acknowledges her feelings of absence, her level of self-awareness increases paradoxically as she directs her attention to the other aspects of her being.

Extract 4c

*“After the breathing exercises, I felt very RELAXED. I was so focused on the breathing and on A.’s voice in my head that I even forgot about the existence of my body and it wasn’t until when A. told us to delicately start moving it [our bodies] that I remembered it again and I started feeling it and connecting with it again. It was a strange feeling but very pleasant and I felt an absolute physical and mental relaxation.” - P6S12*

P6 describes a subjective disconnection from the body during relaxation that she experiences as pleasant. Her narrative suggests a powerful experience *“forgot about the existence of my body”* that is positive to her, probably because she takes a break from the negative thoughts that were mentioned in the previous section.

#### Domain 2: Body and movement

This domain includes participants’ responses to DMT activities that involve the body and its movement. These activities can vary from breathing techniques, becoming aware of the body and exploring different movements with it, individually, in pairs or as a group, with or without materials. (Interpersonal aspects of these activities will be discussed in Domain 3).

**The joys of movement** Participants enjoyed different aspects of the movements in the session.

Extracts 5

*“[...]with the ball, I was able to identify which parts of my body were more receptive.” - P6S05*

A vignette of S05 from the therapist’s diary is presented:

*“ They had their eyes closed and seemed very concentrated on themselves. Their movements were free, direct, sustained and light. I was observing them while they were passing the ball across their body. There was no expression in their faces and I was trying to understand what they were experiencing, what the contact with the object made them feel. I noticed that they would only take care of, touch with the ball, the front part of their body, while ignoring the rear part.”*

*“Later when we had to walk, my fun and “playful” side got out with the ball and the big red cloth (like power).” - P5S10*

A vignette of S10 from the therapist’s diary is presented:

*“ They were all trying the movement proposals. I was very surprised that everyone not only permitted the contact with the other group members but also seemed to enjoy it, laughing and searching different body parts to propose. Seeing that this was well accepted I proposed them to follow the touch of their partners with all of their body. Immediately light, indirect and free movements emerged while the participants were experimenting with the quality of time. When I proposed them to do exactly the opposite, resist to the movement, something completely different happened. They had to put force and use the weight of their body as well as they had to find a minimum grounding. P5 seemed very comfortable and she was enjoying challenging P4. She surprised everyone with what her body was able to do.”*

*“As I was using the rope, at first the space was smaller and more closed, even though with a slit so someone could come near. I liked interacting with the balls, I felt comfortable, integrated and at the same time with the space for myself. I also liked observing how others interacted with each other.” - P2S04*

A vignette of S04 from the therapist’s diary is presented:

*“ Each participant created her own personal space using a woollen thread. They started exploring that space having in general a low tone. I observed light, sustained, indirect and bound movements, while there was no interaction among the participants. In an attempt to facilitate the group coherence I added some balls in the available materials and put some upbeat music. The participants started to interact with each other. At first everyone was lying down and was throwing the balls without using any force. I was mirroring their movements making them progressively bigger and bigger. Suddenly, the participants started to put force in their movements and they were throwing the balls far away, something that made them change from the low to the medium-high level. Their movements were strong, sudden, free and direct. Their bodies seemed bigger, more open and they transmitted a sense of grounding*^2^.”

In the first quote, P6 describes how her sense of body awareness increased by getting in physical contact with an object. P5 describes how she connects with her “playful” side, perhaps a side that she has not been in touch with much since she developed the ED, or even before. P2’s comments experience is an example of how the use of different objects (e.g. ropes, balls) can facilitate the interaction between the participants and also give them a chance to symbolically explore their personal space and limits.

**Difficulties** Several difficulties were mentioned by the participants with regard to the DMT activities.

Extracts 6

*“Sometimes I felt that my body couldn’t move as much as I wanted, that the movements were smaller than I wished.” - P2S05*

*“The part with pushing and using strength, UNCOMFORTABLE. I felt awkward. I couldn’t simply enjoy [the activity] because I didn’t fully let myself go. The cloth was great but since we were holding it between us all, I didn’t feel completely free, to move as I actually needed, to flow.” - P6S06*

*“Today we started with breathing. There I felt nervous.” - P7S10*

*“At the beginning of the session I didn’t feel like doing movements because I felt embarrassed, but as time passed, I started feeling more and more comfortable. Today I listened more to my body and less to my fears.” - P4S06*

*“Later they (my companions) touched me where they believed I needed it, and I touched them. It was very difficult for me to think “where they need it” or “what they need”. And when they touched me, I felt that they didn’t touch me where I needed it, even though I liked it, actually I didn’t know what I needed either.” - P7S12*

*“I missed some music in the movement part. It would have made it easier for me.” -P3S03*

A variety of difficulties are mentioned in these extracts. P2 recognizes her difficulty using her body fully when moving. In the same line, P4 expresses an initial resistance to moving due to feelings of embarrassment that she eventually, at least partially, overcomes. Certain activities and movement qualities tended to be more challenging than others and triggered ambiguous responses, especially the use of strength and breathing exercises. P7 acknowledged her difficulty in recognizing her bodily needs as well as guessing others’. The lack of music was a challenging factor for P3.

**A pattern of activating during the session**

Some of the difficulties mentioned above were overcome during the course of the session as their energy level increased. These extracts were examined in order to identify the helpful elements in the session that triggered a change in the participants’ attitude.

Extracts 7

*“I had two opposite feelings during the session. On the one hand, in the beginning, I was angry and anxious and didn’t want to do anything. It was very difficult to make the movements and the only way for me to be comfortable was on the floor with my face covered. On the other hand, when we started passing the ball I cheered up much more and I even enjoyed it at some point. I think the music cheered me up a lot and helped me.” - P3S04*

*“I couldn’t find a safe place and the blanket that was covering me (imaginary) was bothering me. As I opened my eyes, the feeling diminished. Then we made an activity of using strength, which soothed me very much. I felt liberated, feeling less burdened. The movement made me feel lighter. The tiredness of this week has left me, I feel more active. Now I feel content and motivated to talk and move.” - P7S10*

Analyzing these extracts, it is observed that often the participants who were burdened with negative feelings at the beginning of the session (as presented in theme 1.1.) and without any motivation or energy to move, struggled during the initial self-awareness exercises that required them to be alone with themselves. The key moments that shifted the energy level were identified to happen in pair or group interactions. Moreover, the use of objects like cloths and balls, and of music further facilitated such playful interaction. P7 self-regulates her feelings of anxiety by opening her eyes, and later she finds the activity that requires the use of strength soothing, even though such activities were received more skeptically by some of the participants.

#### **Domain 3: Interpersonal aspects**

Interpersonal aspects were identified as a domain by itself, due to the abundant evidence that the participants presented around this theme.

**Enjoyment of the interactions in the group**

The interactions in the group were identified as an important resource for the participants, in order to raise their energy level. Here they are considered in more detail.

Extracts 8

*“I liked having P7 as my partner. I was surprised positively and it has been a new and different way to get to know her and get a little closer to her.” - P6S10*

*“Later, as P6 was touching me and I had to move, I felt very comfortable and as time passed, we were finding each other more. Finally, we were moving freely but looking at each other, something that made me nervous at first, but in the end, I felt very comfortable and confident.” P5S05*

*“I really liked the next exercise as well. Someone touching my chin, holding it from below. It made me feel especially good, it was like never doubt or never forget to hold your head up high.” - P6S12*

In addition to the increase in the energy level that was mentioned in the previous section, these extracts show the impact of the non-verbal interactions on the participants. P5 describes an intimate moment she found with another participant, as they encountered each other through their movements. P6 describes her enjoyment as she gets in physical contact with the other participants in an exercise focused on giving and receiving care from one another. For her, this encounter has a symbolic meaning of being supported by her companions.

**Difficulties in interpersonal relationships and interaction**

Several challenges and difficulties were mentioned with respect to interactions and relationships, within the group and outside. These issues can be summarized as fear of intimacy, difficulty expressing one’s needs, and preoccupations about what others might think of oneself.

Extracts 9a

*“The thing with eye contact was very intense for me. It is like letting someone in. It wasn’t exactly uncomfortable, but maybe strange, a little violent perhaps.” - P6S05*

*“I also liked interacting with the other person, especially when I was the one being touched, feeling the movement of the other person conveyed more uncertainty because you don’t know where she will take you. I felt more uncomfortable having to move while looking at the other, the movements were less fluent as if they were conditioned.” - P2S05*

*“I didn’t want to let people enter my life just to leave later. I went back to dancing on the terrain, finding my companions without any kind of tie that would bind me to someone. I felt free, liberated.” - P7S09*

Touching another group member was mostly received positively, yet maintaining eye contact with a partner, especially while moving, was challenging for many participants. P6 expresses her discomfort and her words suggest that it is linked to a fear of intimacy “like letting someone in”. P2 also describes the uncertainty she felt concerning “where [the other person] will take [her]”. Similarly, P7, who was new in the group at this point, expresses her distrust of others and reluctance in building meaningful relationships with people “just to leave later”, suggesting an underlying fear of abandonment.

Extracts 9b

*“I didn’t have the strength or energy to look for contact but when it came to me (when the others came near to interact) it made me feel good, even though also a little sad at the same time for not being able to do the same, for having so little energy. - P6S09*

*“[…] I only wanted to feel human contact (...) She [the therapist] told us to get in pairs and it was the last thing I wanted to do, and when I could, I started playing with the balloon alone. I connected with the feeling of loneliness that I have been carrying for a while, usually, it comes up with losses. The best of the session: When P4 came to give me a hug.” - P5S11*

While the participants were eager to give care and received it thankfully, it was difficult for them to ask for it. When P6 receives the contact that she needed, she is not able to enjoy it purely as she is concerned that she cannot offer the same to the others. P5 recognizes her need for contact but she is ambivalent about it and tries to avoid others by playing alone. It is noteworthy that even though these participants did not express their needs openly, in some cases the group sensed them and responded to them.

Extracts 9c

*“Today I found it harder to connect than usual, I don’t think I could really connect, because I was thinking about what others would think of me (that’s why I don’t think the movement part helps me much since I hold myself back.” - P4S03*

*“I felt very comfortable with her, even though I was worried that she didn’t feel the same way” (referring to an exercise in pairs) - P5S10*

*“I am sad, sad because I don’t have P. now [she is referring to her long-distance relationship] and I look for any tiny detail to support my distortion that he doesn’t like me and he will end up getting tired of me (…). I feel sorry that today was the last session and I’ve been so distant [...].” - P5S12*

The first two quotes express the participants’ worries about what others might think about them, whether they will be liked or accepted. This seems to impair their capacity to get the most out of the movement experience. In the last quote, P5 expresses a deep fear of not being loved by her significant other, and while rationally she regards this as her “distortion”, emotionally she seems to be heavily burdened to the point that she is not able to be present in the session.

#### **Domain 4: Metaphors**

According to Lakoff and Johnson [70], metaphor is understood widely as a general function of the mind where a concept is described by its similarity to another, and it includes not only linguistic representations but also dreams, memories, and feelings.

**The inner safe place**

Imaginative techniques were applied frequently in the sessions. In one of the imaginative techniques borrowed from psychodynamic imaginative trauma therapy called the *“inner safe place”* [57], the participants were guided to think of a place, which could be a real place or a fantasy place that conveyed them a sense of safety and security. Most participants wrote about their inner safe place in their journals, often including vivid and detailed descriptions, and they also mentioned the process of looking for their inner safe places and their relationship to it.

Extracts 10a

*“This time my safe place was the beach but without many people and even though it was hot, it wasn’t overwhelming (…). It’s warm and I only feel cold as I’m leaving because the sun has set, but I am happy, feeling the touch of that warmth on my skin.” - P2S07*

*“My place of protection is my room, but with some changes. The window opened to a terrace with a grass floor (it’s night and you can hear the noise of the leaves moved by the wind). Terrace, bed, table, window, grass, night, wind, leaves.(…) Music is heard in the room (…) and cats accompany me. They don’t make a mess and they don’t kill each other.” -P4S08*

*“I can’t see the ocean but it’s inside me. Confusion. A coffeehouse in Paris, empty, and made from dark wood. There are many tables with many white cups, and emptiness in all of them. Paris looks nice…The Eiffel Tower, trees, bicycles, what a cliche…But it is in the exterior. I can observe the landscape through the big windows of the cafe. Calmness.” -P4S09*

While these two participants have different styles describing their safe place, some common elements can be detected in their writings: natural elements (sea, ocean, wind, etc.), emptiness, people or the absence of them. Both descriptions are rich in sensory elements, making the reader imagine these places vividly.

Extracts 10b

*“We imagined a safe place. It wasn’t difficult for me to imagine it at all: the village. It was summer and we were in the village seated on a boat at sunset. It was all silent and he hugged me while we were watching the village. I felt calm, safe and loved.” - P5S07*

*“Today we returned to the safe place but I hate that the village is not my safe place if P. isn’t there, so I imagined another moment, when I was with T. but without focusing on her company.” - P5S08*

*“My safe place turns out to be a person, I realized this today, since the last time we did this exercise, this person was present in my mind again so that I came to realize that it’s not about the place, it’s about the person. E. is my safe place. This scares me and frightens me. Each time we finished and A. told us to slowly say goodbye and that we could always return to it, I felt a tremendous pain because I don’t have this certainty. So my safe place scares me, mostly because I’m afraid to lose it and since I feel that it is so safe and comfortable, that would be very painful.” - P6S07*

In these extracts, the focus of the inner safe place is shifted to another person. P5 enjoys the feeling of being loved by this person, and it seems that she finds it difficult to feel safe when this person is absent. She copes with this by imagining the company of a friend, and thus shifting her focus. Similarly, P6 has difficulties with the exercise of the safe place, when she comes to realize that she is projecting her sense of security onto a person, and she faces her fear of losing this person. In these cases, the safe place technique provided a source of self-knowledge for these participants.

Extracts 10c

*“What I did realize is that as we were given the instructions, I was trying to change or question “my safe place”, like it was difficult to rationally trust my decision” - P1S08*

*“Today I felt confused, impotent and frustrated. My head was running a thousand per hour and I found it hard to focus on my safe place.” - P6S08*

*“In today’s session, I imagined the safe place again. […] I didn’t feel so safe. This made me nervous. So, dancing lightly, I imagined myself dancing on the terrain, making it mine. I felt relieved because it became [safe] again.” - P7S09*

These participants refer to the process of finding their safe place which was not always easy. P1 finds it difficult to maintain her focus on one safe place. P6’s inability to focus her wandering mind gets in the way of accessing her safe place. When P7 does not feel safe, she uses symbolic movements and dance to regain her feeling of safety and she achieves it.

**Metaphorical expressions**

Some participants described their experience via metaphorical expressions.

Extracts 12

*“(…) I remained like the flower in the middle, I haven’t taken everything out, I haven’t completely opened myself. I feel somewhat relieved but not liberated.” - P6S06*

*“What is this mental capsule that I am in? It is so strong, no one can break it. It has a little window at eye level through which I can see. I am protected but it seems like a bad place, unhealthy, an exaggerated cover, or protection.” - P4S11*

In these extracts, the participants do not name the relevant emotions in order to describe their feelings, instead they use metaphors that help the reader get closer to that particular experience. With *“the flower in the middle”*, P6 is referring to a drawing that she made during this session, where she drew three flowers in the process of opening: the first one being closed, the second one half-opened, and the third one being completely opened. The inside vs. outside theme from the safe place can be recognized in P4’s writing again. The strong capsule functions as a defense mechanism, and she recognizes its dysfunctionality (*unhealthy, bad, exaggerated cover*) as well as being protected by it.

#### Domain 5: Reflections

**Reflections on one’s personal process** An evolution in the reflections of the participants was visible when they attended the sessions for the whole period. Below, a collection of extracts from the writings of two patients is presented.

Extracts 13a

*“We started by becoming aware of our bodies through a ball. It made me realize the long path that I have in front of me to recover my weight.” - P5S05*

*“I am more open-minded now because I’ve learned that: If I don’t want to do something (in this case [coming in] neither the clinic nor the sessions), before I wouldn’t do it, but having continued, it’s been good for me to change my mind.” - P5S07*

*“As I am so well outside the center, at home and with P., …I feel that my life starts to flow again. Coming to the clinic makes me feel as if I’m wasting time, since life is passing and I’m still shut-in, without being able to move.” - P5S11*

In these extracts, P5 changes attitude towards the treatment. The movement activity in an earlier session confronts her with her low body weight and makes her think of the “long path” in front of her. It is unclear whether this is a source of despair or motivation. Later, she reveals her skepticism about the treatment at first, both the Clinic in general and the DMT sessions, and that she has changed her mind. In the last quote, she expresses her frustration in the Clinic as she believes that her life is back on track and coming to the Clinic is what holds her back. She uses an observational and cognitively focused style in these extracts.

Extracts 13b

*“Fear, fear, fear. I want to do things and there it is. The present fucked me up. It is okay, maybe fucked up is too much because there is a way out and it is in me.” - P4S10*

*“(…) I don’t feel invisible as I am writing these lines, I don’t feel like I’m hiding, but rather, I’m taking some time for myself, that I am connected with myself and at the same time I am seen, and this causes me some kind of insecurity.” - P4S11*

*“Loneliness. I think that’s what it’s mostly about, next to other things. In the end, the core of all my worries is fear and the base of it loneliness, not feeling cared for, loved, as if I am not worthy of it or don’t deserve it.” P4S12*

These extracts show P4’s process throughout the last sessions as she gets in touch with difficult feelings such as fear and loneliness, and expresses them in her writings. Her expression suggests awareness and authenticity. In the last session, she develops an insight as she links her problems to feelings of worthlessness.

**On the DMT sessions**

As the therapeutic process was coming to an end, some participants included an overall view of the therapy in their reflective diaries, offering a qualitative evaluation of it, and revealing what it meant for them.

Extracts 14

*“(…) these sessions have been very special for me. I was able to connect with myself more and see things or discover thoughts or internal worries that were unknown to me. I had the opportunity to feel closer to my companions and create a new type of connection where words haven’t been necessary. That’s why I’m very thankful to have had the opportunity to do dance therapy with A. - P6S12*

*“Even though I wish we had more sessions, this is the reality and I decide to stay with what I’ve learned, the skills, the moments and emotions. Thank you for accompanying me during such a hard time.” - P4S12*

*“At first I thought this therapy wouldn’t help me, that it was nonsense. It showed me much more than that. It helped me connect with myself, to get to know myself better, where are my limits, how to calm myself down when there are strong feelings. I am very thankful to you A. for all that you’ve achieved in me.” - P5S12*

*“These sessions helped me a lot even though I did it for little time. When I entered demotivated, I left feeling very content and active. When I entered with a lot of anxiety, it helped me decrease it.” - P7S12*

Most participants expressed their thankfulness to the therapist. They reported that the DMT sessions promoted self-awareness, helping them connect with themselves, identify their feelings, and recognize their limits. P4 feels sorry that the therapy did not last longer. Also participants perceived that the sessions reduced their levels of anxiety and gave them energy and motivation. It is noteworthy that P5 attributes the source of change entirely to the therapist: *“all that you’ve achieved in me”*. Finally, the exploration of non-verbal interactions with the peers, the creation of new bonds and the accompaniment of the therapist were appreciated.

## Discussion

The primary aim of this pilot study was to analyze the impact of a DMT intervention on body image and alexithymia in individuals with EDs. Secondly, a qualitative study arm was integrated in order to gain a deeper understanding of the therapeutic process. The following discussion of the quantitative outcomes should be regarded cautiously, as the small sample size does not allow making any generalisations of the obtained results. Instead, they should be taken as a departure point for future studies.

The participants who took part in the DMT group improved on the Appearance Evaluation and Body Areas Satisfaction scales, meaning that they felt more positively about their bodies after the DMT intervention. They scored lower on the Appearance Orientation scale after the therapy, which suggests that they were less preoccupied with their looks. These changes were both statistically and clinically significant. The decrease in the Overweight Preoccupation scale was not statistically significant but a medium to large effect size was observed, suggesting that there have been clinically significant improvements which were not statistically significant due to the small sample size. The control group that continued the treatment as usual did not change significantly in any of the body image aspects measured by the MBSRQ. A mixed-effects ANOVA model showed significant results in the interaction effect for Appearance Orientation and Body Areas Satisfaction. With the necessary caution because of the small sample size, these results suggest that it might be beneficial to include a treatment component to work with individuals on a bodily level in order to address body image issues. Considering that body image is an important maintenance and prognosis factor in EDs [71], helping individuals construct a healthy body image is a crucial element that needs to be covered in any treatment modality.

There is a growing body of evidence suggesting that DMT has positive effects on body image in various populations [49, 50]. Regarding the field of EDs, one study has found DMT to improve body image and self-esteem, and decrease psychological distress, in obese women with emotional eating [51]. However, despite the existence of rich theoretical contributions that dance movement therapists made to the field of EDs [72] and several DMT case studies [73–75], to the authors’ knowledge, no empirical studies evaluated the effects of a DMT intervention in individuals with AN, BN, and EDNOS. Therefore, the present pilot study has been an initial attempt to fill in an important research gap, especially bearing in mind the central role that body image plays in EDs. Hopefully, these promising preliminary results will encourage other researchers to conduct similar studies with larger samples.

The alexithymia scores of the DMT group tended to reduce or remain stable, while there was a tendency to increase in the control group, however these changes were not significant for either group. The stability in alexithymia in the DMT group is not surprising, concerning that the paucity of studies testing effects of diverse psychological treatments on alexithymia in patients with EDs raised diverging results [15, 76]. Even in studies that achieved to reduce alexithymia scores through treatment, the alexithymia levels often remained elevated, so that the clinical significance of these results is unclear [76]. As alexithymia seems to be a trait that is resistant to change, longer treatment periods might be necessary to achieve significant results. On the other hand, the tendency to increase in alexithymia that was observed in the control group was unexpected. While this result should be interpreted with caution due to the small sample size (n = 5 for the control group), it can be an interesting research question whether cognitive behavioral techniques, when these do not specifically target alexithymia, increase alexithymia levels in patients with EDs, as it encourages the patients to take a distant stance from their personal experiences [77].

The TAS-20 was used to measure alexithymia in the present study. While the TAS-20 is a widely used and accepted instrument, one study criticized its use with patients with EDs, as they found that patients with EDs with high alexithymia levels according to TAS-20 performed equally well as the control group on tasks of emotion identifying and reporting. They also found that depression and anxiety states can alter alexithymia scores [78]. While there is no way of knowing if such an effect occurred in the current study, the qualitative analysis has shed more light on alexithymia and related issues in the participants of the DMT group.

The results of the qualitative analysis demonstrated difficulties in identifying feelings and a difficulty to be present in the moment. Some participants reacted defensively when they encountered strong feelings during the sessions. However, in some instances, they were able to face and accept these strong feelings. The DMT sessions seem to have provided a secure base for the participants to explore such feelings. In their overall evaluations of the intervention, participants reported that the sessions helped them be more present in the moment, identify their feelings, and get to know themselves better. Even though no significant results were obtained in the TAS-20 scores, these patient accounts suggest that the DMT sessions had an impact on some aspects of alexithymia.

Specific attention was given to the use of metaphors as patients’ with EDs impaired symbolic thinking and their tendency to somatize their emotional experience have been identified as a challenge to traditional psychotherapy [79]. The use of rich metaphors by some patients and their vivid descriptions of their “safe place” contradict with the impoverished inner fantasy life that is associated with alexithymia [19]. The work with active imagination in movement is a core aspect of DMT. This process allows the patient to work with metaphors and symbolism, thus may help the patient access contents that were previously not conscious. These results suggest that the use of imagery techniques and creative movement in DMT, while putting an emphasis on body awareness, can help combat these limitations.

The participants reported that the intervention had a positive effect on their mood, helping them feel more cheerful or motivated, and less anxious, confused or angry. These qualitative results are in line with a previous study using self-report questionnaires that revealed an increase in energy and decrease in anger and confusion in adolescents in a day hospital after the DMT sessions [80]. In the present study, the extracts from the patients’ diaries also shed light on how the DMT sessions facilitated such change in mood. Most participants described a similar pattern of entering in the session in a depressed or anxious mood and initially had difficulties engaging in the activities. The therapist accepted and attuned to their initial mood while gently encouraging them to explore different movements. Furthermore, the use of music and materials such as balls and cloths facilitated playful interaction between the group members and raised their energy level. These effects can be related to neurohormonal effects of movement, as a previous study observed changes in dopamine and serotonin levels as well as a decrease in psychological distress after a 12-week DMT intervention in middle school students [81].

However, it is important to note that such effects can only partially be explained by the benefits of physical activity, as one study comparing a single circle dance intervention to exercise on a home trainer bike found that depression levels decreased in the dance condition and not in the exercise condition [82]. Dancing in a group seems to have more components that promote mental health than physical exercise only, like the physical and affective interaction between group members. What is more, physical exercise is a complicated issue in the treatment of EDs [83] as patients often use excessive exercise for complex psychological reasons [84]. DMT could provide an alternative to traditional physical exercise, since it seems to have the potential to promote body awareness, as well as benefit the body and mental health on a neurohormonal level. This would be valuable for ED treatment, as patients often suffer from comorbidities with depression and anxiety [85]. Larger scale studies including biomarkers are needed in order to test these assumptions for patients with EDs.

Some interpersonal difficulties that are associated with EDs according to a recent systematic review [86], have emerged in the participants’ reflective diaries, such as interpersonal distrust, fear of intimacy, and difficulty expressing one’s needs. At the same time, the empathetic interactions in the group, both with each other and with the therapist, were valued by the group members, similar to findings in a cognitive behavioral therapy group [87]. A novelty in this study is the focus on the nonverbal aspect of these interactions. The reflective diaries showed clearly that the participants enjoyed getting to know one another on verbal and nonverbal levels. At the same time, some of the DMT techniques prompted them to reflect on their real-life relationships. Bearing in mind the importance of positive social relationships in the recovery of EDs [71], and the various interpersonal problems that they often suffer from [86], attending the interpersonal aspects has a crucial role in ED treatment. DMT can be an innovative way to address these issues, as it combines implicit and explicit learning, verbal and nonverbal interaction.

While the group format seems to have helped the participants enhance in relationship dimensions, it should be noted that individual DMT sessions would have its own advantages, such as the patients having more space to explore their movements freely. The privacy and confidentiality that individual therapy offers could allow patients to feel more secure and talk about personal issues in depth.

Interestingly, the participants made few references to body image issues in their reflective diaries. This might be because they were more focused on their lived bodies during the DMT sessions, and not so much on their appearances. It also suggests that the improvements in body image found in the MBSRQ scores happened through implicit ways. For instance, the writings indicate a pure enjoyment of the playful social interactions that the participants experienced in the sessions. Considering that patients with EDs tend to experience their bodies through external means such as weighing scales, mirrors, etc. [79], the enjoyment through the lived body that DMT offers can be very valuable and this might be accountable for the improvement in body image found by the MBSRQ. This implicit aspect marks a significant difference between DMT and approaches that put an explicit focus on body image issues, such as cognitive-behavioral techniques. Lastly, most participants expressed their gratitude to the therapist in their reflective diaries. Overall, they were satisfied with the intervention proposed. There were no dropouts in the DMT group and the no-show rates were low, suggesting that the patients were committed to the therapy. This seems important considering the high dropout rates in EDs [88].

### Limitations

The findings of this pilot study have to be considered in light of some important limitations. As authors such as Schweizer and Furley emphasize, prior to any research the appropriate sample size should be calculated in order to obtain more accurate, reliable and reproducible results. However, these authors also recognize that in certain circumstances this could make research difficult in certain areas of knowledge in which access to a sufficiently large sample is limited by numerous factors [89].

The authors of the present study are extremely cautious about the quantitative results obtained, as Button et al. suggest. They consider the choice of a methodological design such as the mixed one to be an ethical procedure. Given the difficulty of having a sufficiently large sample, this makes it possible to incorporate other data collection and analysis tools that offer information of interest in relation to the subject matter being dealt with [90].

Another issue that compromises the reliability of the quantitative results is the heterogeneity between the groups. The intervention and the control groups were homogeneous concerning their age and BMI, however the control group had been in the Clinic for a considerably longer period than the DMT group. A larger sample would be necessary to determine the influence that the time spent in the Clinic had on the results. Furthermore, the sample was heterogeneous concerning the ED subtype, with the majority in both conditions being EDNOS or AN, and only one BN patient in the control group. Therefore, it remains unknown if the diagnosis of a particular ED subtype had an effect on how the DMT sessions were received. The sessions were conducted by a DMT trainee, who was doing her clinical internship in the Clinic. She was at the moment in the beginning of her learning process and had no previous experience with this population. A therapist with more experience might have worked more efficiently and adapt better to the particularities of each participant or the ones of the group.

Another shortcoming was that the DMT group was a semi-open group. For the therapeutic process this implies working with individuals who can be in different moments of their personal process, which might present an additional challenge to the group therapist. A closed group would be beneficial for the patients in terms of developing trust in the group and getting used to working with their bodies. In terms of methodological rigor the variation in the number of sessions that each participant attended to, poses another variable that can not be controlled. The setting of the DMT group was adjusted to the needs of the private clinic where the study was conducted. In line with the Clinic’s policies, two interns attended as observers the majority of the sessions, which presented a confounding variable that might have affected the results. Finally, this was an all-Spanish sample, and some interventions might not work in the same way with patients from other cultures, e.g. interventions that include touch and eye contact, as these aspects of nonverbal communication vary significantly across cultures. Practitioners should take the cultural context into account.

To the best of the authors’ knowledge, there are no quantitative studies evaluating the impact of DMT interventions on the body image and alexithymia rates of patients with EDs. The authors had no reference point when designing this pilot study. The findings are promising despite the mentioned limitations, as significant improvements were observed in body image, and a slight tendency for improvement in alexithymia, compared to the control group. It would be interesting to be able to follow this line of research with larger samples which would minimise statistical analysis limitations. Last but not least, it should be taken into account that in bodywork approaches like DMT, a large sample does not make it possible to do in-depth work. In any case, mixed methodological research design is crucial, since the qualitative information collected from the patients themselves offers a better understanding of the process by giving space to each of their individual experiences. The integration of both approaches, quantitative and qualitative, should be, in the authors’ opinion, the optimal way to proceed in this field.

## Conclusions

To the authors’ knowledge, the present pilot study is the first controlled trial to evaluate the impact of a DMT intervention on body image and alexithymia in a mixed group of patients with EDs, i.e., AN and EDNOS. Furthermore, the study has provided valuable insights into the experience of patients with EDs, using qualitative analysis of the reflective diaries written by the participants at the end of each session. The DMT group showed significant improvement in various body image scales, while there were no significant changes in the alexithymia scores. Moreover, the qualitative analysis revealed other factors of the DMT sessions that were appreciated by the participants, as they reported an increase in self-awareness, improvement in emotional states, as well as they valued verbal and non-verbal interaction with the group members and with the therapist. More research is necessary to confirm these promising findings. Combined, these results reveal the potentially significant impact that a DMT intervention might have in the treatment of patients with EDs. The combination of DMT with the traditionally proposed ED treatments might provide a more holistic and effective approach.

## Data Availability

The quantitative data set and the full qualitative analysis including quotes may be requested from the corresponding author upon reasonable request.

## Abbreviations

(ED): Eating disorder
(DMT): Dance and movement therapy
(TAS-20): Toronto alexithymia scale
(DSM-5): Diagnostic and statistical manual of mental disorders, fifth edition
(MBSRQ): Multidimensional body-self relations questionnaire
(SPSS): Statistical package for the social sciences

## Footnotes

1 A. pseudo-initial for the therapist.

2 The references in movement qualities, from the therapist’s diary, derived from Laban movement analysis that is used in DMT. They are related to the indulgence or the resistance of the participant and may indicate the way of relating with the other.
